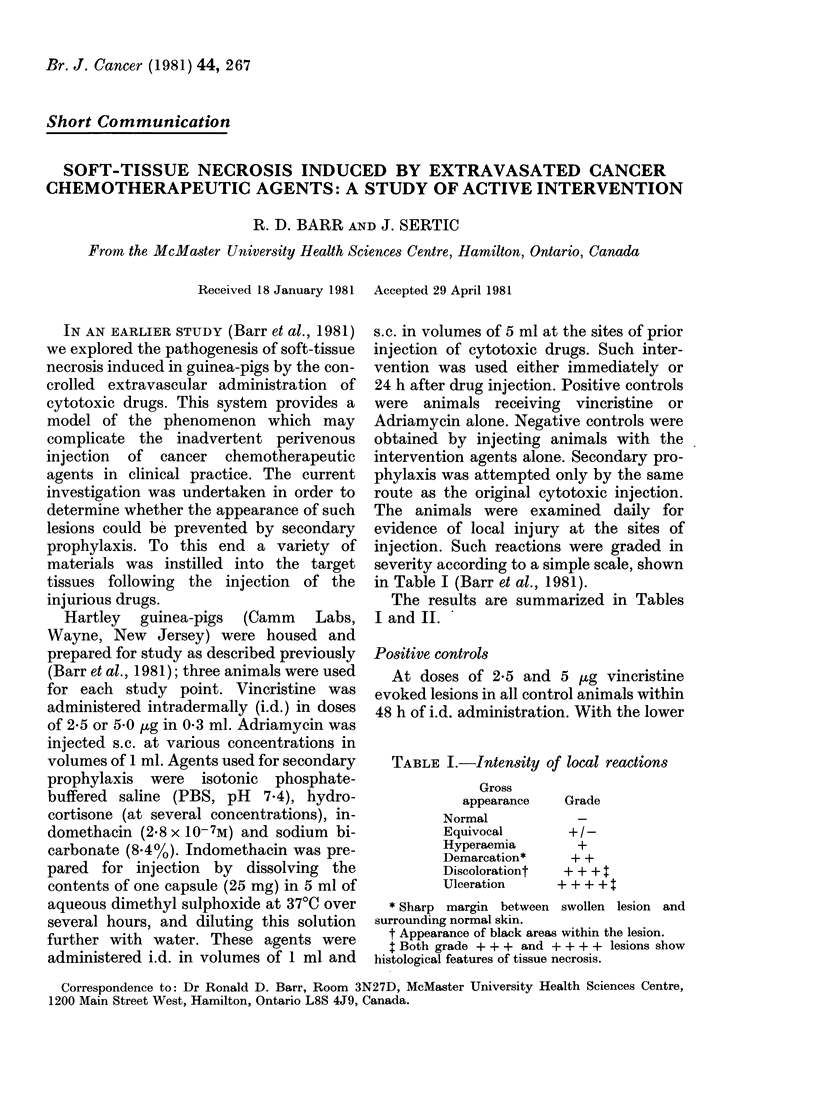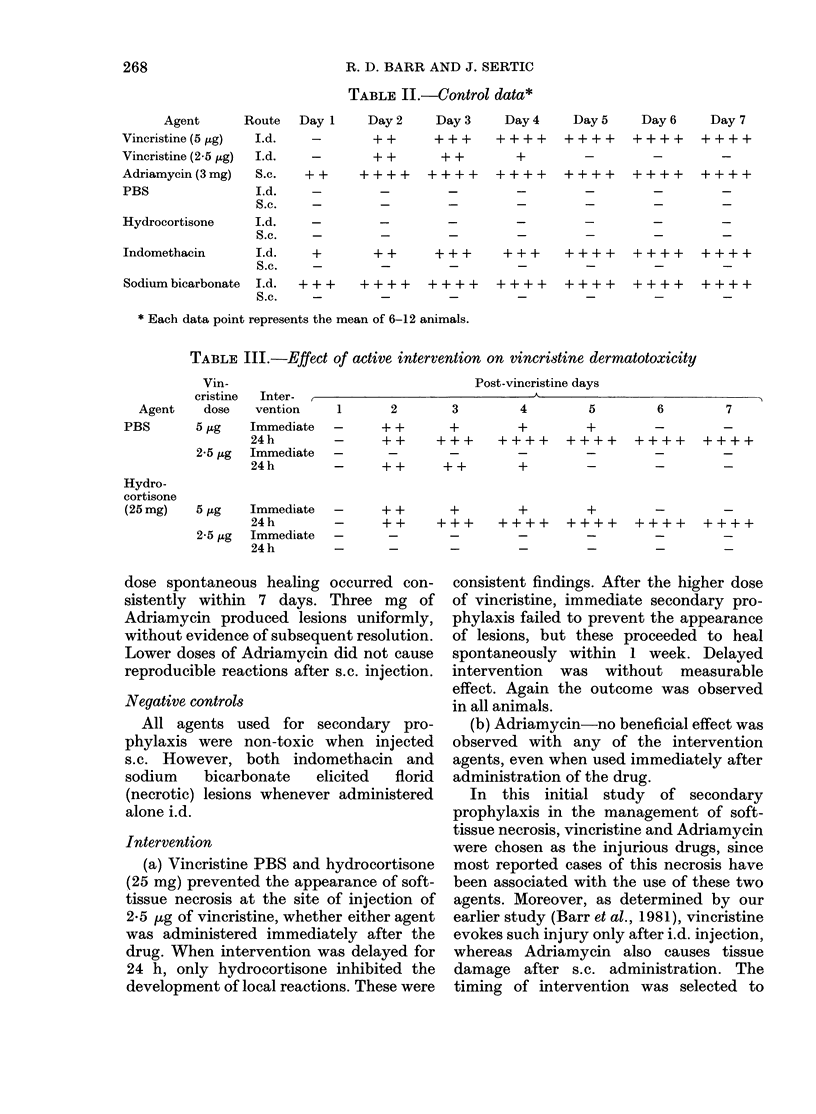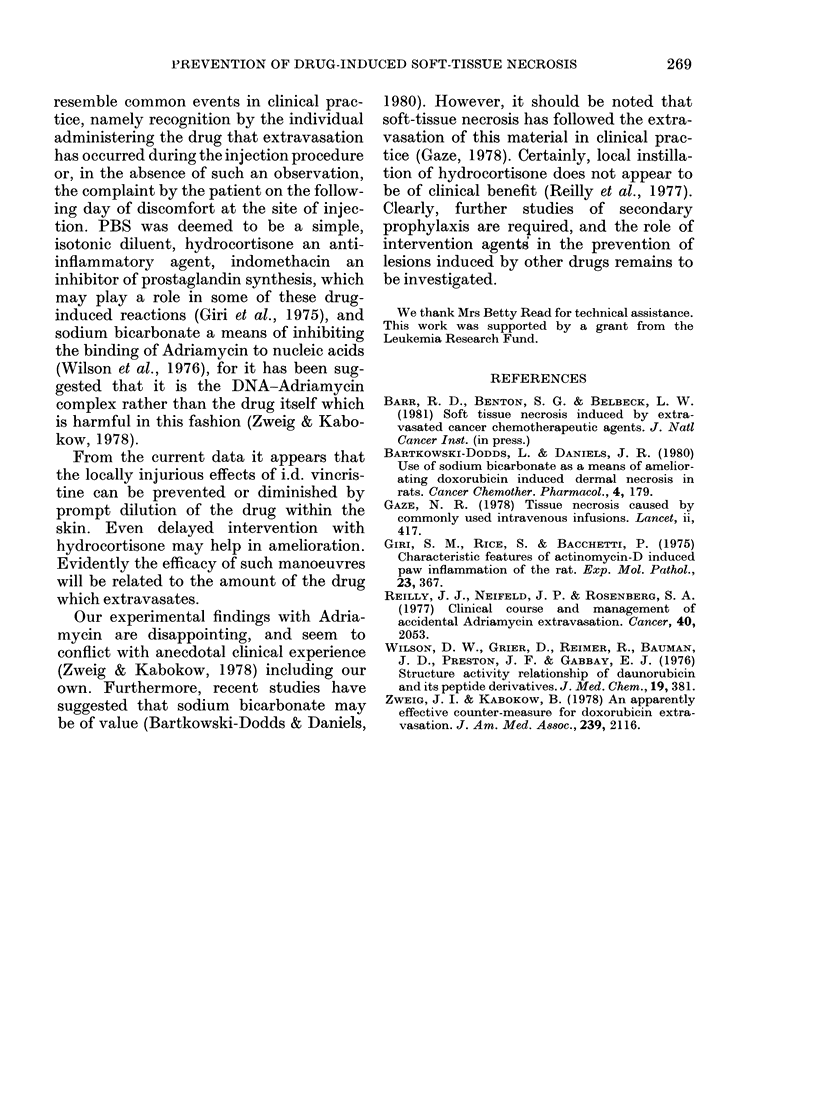# Soft-tissue necrosis induced by extravasated cancer chemotherapeutic agents: a study of active intervention.

**DOI:** 10.1038/bjc.1981.179

**Published:** 1981-08

**Authors:** R. D. Barr, J. Sertic


*Br. J. Cancer* (1981) **44**, 267

### *Short Communication*

### SOFT-TISSUE NECROSIS INDUCED BY EXTRAVASATED CANCER CHEMOTHERAPEUTIC AGENTS: A STUDY OF ACTIVE INTERVENTION

R. D. BARR AND J. SERTIC

*From the McMaster University Health Sciences Centre, Hamilton, Ontario, Canada*

Received 18 January 1981 Accepted 29 April 1981

IN AN EARLIER STUDY (Barr *et al.*, 1981) we explored the pathogenesis of soft-tissue necrosis induced in guinea-pigs by the concrolled extravascular administration of cytotoxic drugs. This system provides a model of the phenomenon which may complicate the inadvertent perivenous injection of cancer chemotherapeutic agents in clinical practice. The current investigation was undertaken in order to determine whether the appearance of such lesions could be prevented by secondary prophylaxis. To this end a variety of materials was instilled into the target tissues following the injection of the injurious drugs.

Hartley guinea-pigs (Camm Labs, Wayne, New Jersey) were housed and prepared for study as described previously (Barr *et al.*, 1981); three animals were used for each study point. Vincristine was administered intradermally (i.d.) in doses of 2·5 or 5·0 μg in 0·3 ml. Adriamycin was injected s.c. at various concentrations in volumes of 1 ml. Agents used for secondary prophylaxis were isotonic phosphate-buffered saline (PBS, pH 7·4), hydrocortisone (at several concentrations), indomethacin ($2{\cdot}8 \times 10^{-7}$M) and sodium bicarbonate (8·4%). Indomethacin was prepared for injection by dissolving the contents of one capsule (25 mg) in 5 ml of aqueous dimethyl sulphoxide at 37°C over several hours, and diluting this solution further with water. These agents were administered i.d. in volumes of 1 ml and s.c. in volumes of 5 ml at the sites of prior injection of cytotoxic drugs. Such intervention was used either immediately or 24 h after drug injection. Positive controls were animals receiving vincristine or Adriamycin alone. Negative controls were obtained by injecting animals with the intervention agents alone. Secondary prophylaxis was attempted only by the same route as the original cytotoxic injection. The animals were examined daily for evidence of local injury at the sites of injection. Such reactions were graded in severity according to a simple scale, shown in Table I (Barr *et al.*, 1981).

The results are summarized in Tables I and II.

### *Positive controls*

At doses of 2·5 and 5 μg vincristine evoked lesions in all control animals within 48 h of i.d. administration. With the lower

TABLE I.—*Intensity of local reactions*

| Gross appearance | Grade |
|---|---|
| Normal | – |
| Equivocal | +/– |
| Hyperaemia | + |
| Demarcation* | + + |
| Discoloration† | + + + ‡ |
| Ulceration | + + + + ‡ |

* Sharp margin between swollen lesion and surrounding normal skin.

† Appearance of black areas within the lesion.

‡ Both grade + + + and + + + + lesions show histological features of tissue necrosis.

Correspondence to: Dr Ronald D. Barr, Room 3N27D, McMaster University Health Sciences Centre, 1200 Main Street West, Hamilton, Ontario L8S 4J9, Canada.

TABLE II.—*Control data**

| Agent | Route | Day 1 | Day 2 | Day 3 | Day 4 | Day 5 | Day 6 | Day 7 |
|---|---|---|---|---|---|---|---|---|
| Vincristine (5 μg) | I.d. | − | ++ | +++ | ++++ | ++++ | ++++ | ++++ |
| Vincristine (2·5 μg) | I.d. | − | ++ | ++ | + | − | − | − |
| Adriamycin (3 mg) | S.c. | ++ | ++++ | ++++ | ++++ | ++++ | ++++ | ++++ |
| PBS | I.d. | − | − | − | − | − | − | − |
| | S.c. | − | − | − | − | − | − | − |
| Hydrocortisone | I.d. | − | − | − | − | − | − | − |
| | S.c. | − | − | − | − | − | − | − |
| Indomethacin | I.d. | + | ++ | +++ | +++ | ++++ | ++++ | ++++ |
| | S.c. | − | − | − | − | − | − | − |
| Sodium bicarbonate | I.d. | +++ | ++++ | ++++ | ++++ | ++++ | ++++ | ++++ |
| | S.c. | − | − | − | − | − | − | − |

* Each data point represents the mean of 6–12 animals.

TABLE III.—*Effect of active intervention on vincristine dermatotoxicity*

| Agent | Vincristine dose | Intervention | Post-vincristine days 1 | 2 | 3 | 4 | 5 | 6 | 7 |
|---|---|---|---|---|---|---|---|---|---|
| PBS | 5 μg | Immediate | − | ++ | + | + | + | − | − |
| | | 24 h | − | ++ | +++ | ++++ | ++++ | ++++ | ++++ |
| | 2·5 μg | Immediate | − | − | − | − | − | − | − |
| | | 24 h | − | ++ | ++ | + | − | − | − |
| Hydrocortisone (25 mg) | 5 μg | Immediate | − | ++ | + | + | + | − | − |
| | | 24 h | − | ++ | +++ | ++++ | ++++ | ++++ | ++++ |
| | 2·5 μg | Immediate | − | − | − | − | − | − | − |
| | | 24 h | − | − | − | − | − | − | − |

dose spontaneous healing occurred consistently within 7 days. Three mg of Adriamycin produced lesions uniformly, without evidence of subsequent resolution. Lower doses of Adriamycin did not cause reproducible reactions after s.c. injection.

*Negative controls*

All agents used for secondary prophylaxis were non-toxic when injected s.c. However, both indomethacin and sodium bicarbonate elicited florid (necrotic) lesions whenever administered alone i.d.

*Intervention*

(a) Vincristine PBS and hydrocortisone (25 mg) prevented the appearance of soft-tissue necrosis at the site of injection of 2·5 μg of vincristine, whether either agent was administered immediately after the drug. When intervention was delayed for 24 h, only hydrocortisone inhibited the development of local reactions. These were consistent findings. After the higher dose of vincristine, immediate secondary prophylaxis failed to prevent the appearance of lesions, but these proceeded to heal spontaneously within 1 week. Delayed intervention was without measurable effect. Again the outcome was observed in all animals.

(b) Adriamycin—no beneficial effect was observed with any of the intervention agents, even when used immediately after administration of the drug.

In this initial study of secondary prophylaxis in the management of soft-tissue necrosis, vincristine and Adriamycin were chosen as the injurious drugs, since most reported cases of this necrosis have been associated with the use of these two agents. Moreover, as determined by our earlier study (Barr *et al.*, 1981), vincristine evokes such injury only after i.d. injection, whereas Adriamycin also causes tissue damage after s.c. administration. The timing of intervention was selected to resemble common events in clinical practice, namely recognition by the individual administering the drug that extravasation has occurred during the injection procedure or, in the absence of such an observation, the complaint by the patient on the following day of discomfort at the site of injection. PBS was deemed to be a simple, isotonic diluent, hydrocortisone an anti-inflammatory agent, indomethacin an inhibitor of prostaglandin synthesis, which may play a role in some of these drug-induced reactions (Giri *et al.*, 1975), and sodium bicarbonate a means of inhibiting the binding of Adriamycin to nucleic acids (Wilson *et al.*, 1976), for it has been suggested that it is the DNA–Adriamycin complex rather than the drug itself which is harmful in this fashion (Zweig & Kabokow, 1978).

From the current data it appears that the locally injurious effects of i.d. vincristine can be prevented or diminished by prompt dilution of the drug within the skin. Even delayed intervention with hydrocortisone may help in amelioration. Evidently the efficacy of such manoeuvres will be related to the amount of the drug which extravasates.

Our experimental findings with Adriamycin are disappointing, and seem to conflict with anecdotal clinical experience (Zweig & Kabokow, 1978) including our own. Furthermore, recent studies have suggested that sodium bicarbonate may be of value (Bartkowski-Dodds & Daniels, 1980). However, it should be noted that soft-tissue necrosis has followed the extravasation of this material in clinical practice (Gaze, 1978). Certainly, local instillation of hydrocortisone does not appear to be of clinical benefit (Reilly *et al.*, 1977). Clearly, further studies of secondary prophylaxis are required, and the role of intervention agents in the prevention of lesions induced by other drugs remains to be investigated.

We thank Mrs Betty Read for technical assistance. This work was supported by a grant from the Leukemia Research Fund.

### REFERENCES

Barr, R. D., Benton, S. G. & Belbeck, L. W. (1981) Soft tissue necrosis induced by extravasated cancer chemotherapeutic agents. *J. Natl Cancer Inst.* (in press.)

Bartkowski-Dodds, L. & Daniels, J. R. (1980) Use of sodium bicarbonate as a means of ameliorating doxorubicin induced dermal necrosis in rats. *Cancer Chemother. Pharmacol.*, **4**, 179.

Gaze, N. R. (1978) Tissue necrosis caused by commonly used intravenous infusions. *Lancet*, ii, 417.

Giri, S. M., Rice, S. & Bacchetti, P. (1975) Characteristic features of actinomycin-D induced paw inflammation of the rat. *Exp. Mol. Pathol.*, **23**, 367.

Reilly, J. J., Neifeld, J. P. & Rosenberg, S. A. (1977) Clinical course and management of accidental Adriamycin extravasation. *Cancer*, **40**, 2053.

Wilson, D. W., Grier, D., Reimer, R., Bauman, J. D., Preston, J. F. & Gabbay, E. J. (1976) Structure activity relationship of daunorubicin and its peptide derivatives. *J. Med. Chem.*, **19**, 381.

Zweig, J. I. & Kabokow, B. (1978) An apparently effective counter-measure for doxorubicin extravasation. *J. Am. Med. Assoc.*, **239**, 2116.